# O papel do enfermeiro na prevengo do delirium no paciente adulto/idoso crítico*

**DOI:** 10.15649/cuidarte.1983

**Published:** 2022-10-15

**Authors:** Cláudia Oliveira, Cátia Filipa Garnacho Martins Nobre, Rita Margarida Dourado Marques, Maria Manuela Madureira Lebre Mendes, Patricia Cruz Pontífice Sousa

**Affiliations:** 1 . Universidade Católica Portuguesa, Lisboa, Portugal. Email: claudia.gameiro@hotmail.com Universidade Católica Portuguesa Universidade Católica Portuguesa Lisboa Portugal claudia.gameiro@hotmail.com; 2 . Universidade Católica Portuguesa, Lisboa, Portugal. Email: catiamnobre@gmail.com Universidade Católica Portuguesa Universidade Católica Portuguesa Lisboa Portugal catiamnobre@gmail.com; 3 . Universidade Católica Portuguesa, Lisboa, Portugal. Email: ritamdmarques@gmail.com Universidade Católica Portuguesa Universidade Católica Portuguesa Lisboa Portugal ritamdmarques@gmail.com; 4 . Universidade Católica Portuguesa, Lisboa, Portugal. Email: madureira@ics.lisboa.ucp.pt Universidade Católica Portuguesa Universidade Católica Portuguesa Lisboa Portugal madureira@ics.lisboa.ucp.pt; 5 . Universidade Católica Portuguesa, Lisboa, Portugal. Email: patriciaps@ics.lisboa.ucp.pt Universidade Católica Portuguesa Universidade Católica Portuguesa Lisboa Portugal patriciaps@ics.lisboa.ucp.pt

**Keywords:** Delírio, Prevencáo & Controle, Papel do Profissional de Enfermagem, Enfermagem de Cuidados Críticos, Unidade de Terapia Intensiva, Delirium, Prevention & Control, Nurse's Role, Critical Care Nursing, Intensive Care Units, Delirio, Prevención y Control, Rol de la Enfermera, Enfermería de Cuidados Críticos, Unidades de Cuidados Intensivos

## Abstract

**Introdujo:**

Delirium é uma disfundo cerebral aguda, associado ao aumento da mortalidade e morbilidade, que atinge frequentemente o paciente adulto/idoso crítico. O enfermeiro tem um papel determinante na prevencáo/controlo do delirium, através da implementacáo de intervendes náo farmacológicas.

**Objetivo:**

Conhecer as intervendes de enfermagem na identificado, prevendo e controlo do delirium no paciente adulto/idoso crítico.

**Materiais e Métodos:**

Realizada uma Revisáo Integrativa da Literatura de artigos publicados entre 2014 e 2018, que identificaram intervendes de enfermagem dirigidas a prevendo e controlo do delirium no paciente adulto/idoso crítico. Foram realizadas quatro pesquisas, nas bases de dados electrónicas da EBSCOhost e na B-on.

**Resultados:**

Identificaram-se 13 estudos, que apresentam intervendes de enfermagem, maioritariamente náo farmacológicas, para prevendo e controlo do delirium no paciente adulto/idoso crítico. Destas, evidenciam-se intervendes relacionadas com o ambiente, promodo do sono, intervendo terapéutica precoce, avaliacáo cognitiva e orientado dos pacientes, intervencóes sistematizadas em protocolos, bem como intervendes direcionadas a participado dos familiares, a formado dos enfermeiros e ao ensino dos pacientes. Foram também identificados fatores de risco para o desenvolvimento do delirium e instrumentos de avaliacáo.

**Discussao:**

A prevendo do delirium é importante e imperativa, já que nos pacientes críticos a sua ocorréncia está associada ao aumento da mortalidade, morbilidade, do tempo de internamento e a um elevado custo hospitalar. A identificacáo dos fatores de risco para a ocorréncia do delirium devem estar incluídos nos protocolos de abordagem do delirium.

**Conclusao:**

As evidéncias demonstraram que o enfermeiro é fundamental na identificacáo precoce, prevencáo e controlo do delirium, evitando a progressáo da doenca, contribuindo para a diminuicáo da morbilidade e mortalidade. A intervencáo de enfermagem deve incluir a identificacáo de fatores predisponentes e/ou precipitantes de modo a contribuir para a diminuicáo da ocorréncia e/ou resolucáo do quadro de delirium.

## Abstract (en)

**Introduction:**

Delirium is an acute brain dysfunction, associated with increased mortality and morbidity, which often affects critically ill adult/elderly patients. Nurses have a crucial role in the prevention/control of delirium, through the implementation of non-pharmacological interventions.

**Objective:**

To know the nursing interventions in the identification, prevention and control of delirium in adult/critical elderly patients.

**Materials and Methods:**

An Integrative Literature Review of articles published between 2014 and 2018 was carried out, which identified nursing interventions aimed at the prevention and control of delirium in adult/ critical elderly patients. Four searches were carried out, in the electronic databases of EBSCOhost and B-on.

**Results:**

13 studies were identified, which present nursing interventions, mostly non-pharmacological, for the prevention and control of delirium in adult/critical elderly patients. Of these, interventions related to the environment, sleep promotion, early therapeutic intervention, cognitive assessment and patient orientation, interventions systematized in protocols, as well as interventions aimed at the participation of family members, the training of nurses and the teaching of patients are evident. Risk factors for the development of delirium and assessment tools were also identified.

**Discussion:**

The prevention of delirium is important and imperative, since its occurrence in critically ill patients is associated with increased mortality, morbidity, length of stay and a high hospital cost. The identification of risk factors for the occurrence of delirium should be included in delirium management protocols.

**Conclusions:**

Evidence has shown that nurses are essential in the early identification, prevention and control of delirium, preventing the progression of the disease, contributing to the reduction of morbidity and mortality. The nursing intervention must include the identification of predisposing and/or precipitating factors to contribute to the reduction of the occurrence and/or resolution of delirium.

## Resumen

**Introducción:**

El delirio es una disfunción cerebral aguda, asociada con un aumento de la mortalidad y la morbilidad, que afecta con frecuencia a pacientes adultos/ancianos críticamente enfermos. Las enfermeras tienen un papel crucial en la prevención/control del delirio, a través de la implementación de intervenciones no farmacológicas.

**Objetivo:**

Conocer las intervenciones de enfermería en la identificación, prevención y control del delirio en pacientes adultos/ancianos críticos.

**Materiales y Métodos:**

Se realizó una Revisión Integrativa de la Literatura de artículos publicados entre 2014 y 2018, que identificaron intervenciones de enfermería dirigidas a la prevención y control del delirio en pacientes adultos/ancianos críticos. Se realizaron cuatro búsquedas, en las bases de datos electrónicas de EBSCOhost y B-on.

**Resultados:**

Se identificaron 13 estudios que presentan intervenciones de enfermería, en su mayoría no farmacológicas, para la prevención y control del delirio en pacientes adultos/ ancianos críticos. De estas, se evidencian intervenciones relacionadas con el medio ambiente, la promoción del sueño, la intervención terapéutica temprana, la evaluación cognitiva y la orientación del paciente, intervenciones sistematizadas en protocolos, así como intervenciones dirigidas a la participación de los familiares, la formación de enfermeras y la enseñanza de los pacientes. También se identificaron factores de riesgo para el desarrollo de delirio y herramientas de evaluación.

**Discusión:**

La prevención del delirio es importante e imperativa, ya que su ocurrencia en pacientes críticos se asocia con aumento de la mortalidad, morbilidad, estancia hospitalaria y alto costo hospitalario. La identificación de factores de riesgo para la aparición de delirio debe incluirse en los protocolos de manejo del delirio.

**Conclusión:**

La evidencia ha demostrado que los enfermeros son esenciales en la identificación temprana, prevención y control del delirio, previniendo la progresión de la enfermedad, contribuyendo para la reducción de la morbimortalidad. La intervención de enfermería debe incluir la identificación de factores predisponentes y/o precipitantes para contribuir a la reducción de la ocurrencia y/o resolución del delirio.

## Introdujo

A definigáo de Delirium está associada a uma disfungáo cerebral aguda que pode ser caracterizada pela perturbado da atengáo e da consciencia, de forma transitória e flutuante, acompanhada de uma mudanza na cognigáo basal, e que surge frequentemente em pacientes internados em Unidades de Terapia Intensiva (UTI)^1^^-^^2^.

De acordo com o Manual Diagnóstico e Estatístico de Transtornos Mentais, o delirium costuma estar associado a perturbado no sono-vigília, o que pode incluir sonolencia diurna, agitado noturna, dificuldade em adormecer, sono excessivo durante o dia ou vigília durante a noite. Por vezes pode resultar em inversáo total do sono-vigília/ noite-dia. O indivíduo com delirium pode mostrar perturbagóes emocionais, como ansiedade, medo, depressáo, irritabilidade, raiva, euforia e apatia bem como, mudangas rápidas e imprevisíveis de um estado emocional a outro, o que pode ficar evidente ao chamar, gritar, murmurar, queixar-se ou produzir outros sons. Esses comportamentos sáo especialmente prevalentes a noite e sob condigóes em que faltam estímulos ambientais^2^.

Os critérios de diagnóstico desta perturbagáo sáo cinco: perturbagáo da atengáo e da consciencia; quando se desenvolve num período breve de tempo (horas a dias) e quando há alteragáo e oscilagáo da atengáo e da consciencia basais ao longo do dia; perturbagáo da cognigáo; perturbagáo da percegáo que náo é explicada por demencia pré-existente estabelecida ou em evolugáo; quando há evidencia de que a perturbagáo é resultante de causas fisiológicas devido a uma condigáo médica de múltipla etiologia, diagnosticada através da história, exame físico ou achados laboratoriais^2^.

O delirium pode ser classificado de agudo quando a duragáo é de poucas horas a dias ou de persistente com a duragáo de semanas ou meses. Pode, ainda, ser classificado de hiperativo, hipoativo e misto. No primeiro verifica-se um nível hiperativo de atividade psicomotora, frequentemente com oscilagáo do humor, agitagáo e recusa de cuidados médicos; no segundo constata-se uma hipoatividade e que pode ser acompanhada de lentidáo e letargia que se aproxima do estupor, e no terceiro verifica-se uma alternancia entre os níveis hipoativo e hiperativo^2^.

O delirium está associado a um maior tempo de ventilagáo mecánica, com maior tempo de permanencia na UTI e maior risco de mortalidade. Alguns dos fatores de risco incluem idade avangada, alcoolismo, alteragóes da visáo/audigáo e, no caso de paciente crítico, o uso de contengáo física, dor prolongada e algum tipo de medicagáo^3^.

As intervengóes de enfermagem devem ser ajustadas a cada paciente, tendo em conta a sua individualidade, o seu diagnóstico e as suas preferencias^4^.

Deste modo, torna-se crucial detetar na anamnese fatores de risco e utilizar escalas de avaliagáo validadas tal como a Confusion Assessment Method-ICU (CAM-ICU), que se encontra validada para portugues^5^ e a escala CAM (Confusion Assessment Method) validada para a populagáo portuguesa^6^. De entre os fatores de risco para o delírium, alguns podem ser modificáveis^1^, tais como, a permanencia no leito, as alteragóes hidroeletrolíticas, a hipoxia e a utilizagáo de dispositivos médicos invasivos. O enfermeiro tem um papel preponderante na sua prevengáo através da mobilizagáo precoce, a corregáo de distúrbios hidroelectrolíticos, prevengáo da hipoxia, suspensáo precoce da ventilagáo mecánica e remogáo de dispositivos invasivos. Torna-se por isso importante promover o conforto e o controle da dor, a utilizagáo da menor sedagáo possível, estratégias proativas de desmame da ventilagáo mecánica e início precoce de terapia ocupacional e fisioterapia.

A PADIS (Clinical Practice Guidelines for the Prevention and Management of Pain, Agitation/Sedation, Delirium, Immobility, and Sleep Disruption in Adult Patients in the ICU) recomendada pela Society of Critical Care Medicine's, em 2018, define os fatores de risco como modificáveis (transfusóes de sangue e uso de benzodiazepinas) e nao modificáveis (idade, demencia, coma prévio, cirurgia pré- internamento e índices de gravidade elevados), com forte evidencia da relagáo deste fatores com a ocorrencia do delirum^7^.

O enfermeiro tem por isso um papel muito importante na prevengáo e controlo do delirium. Para uma decisáo clínica e intervengo de enfermagem ajustada, é fundamental uma correta avaliagáo da pessoa/situagáo clínica, um planeamento adequado das intervengóes, a implementagáo dessas intervengóes (farmacológicas e náo farmacológicas) e uma correta reavaliagáo.

Foi definido como objetivo desta RIL (Revisáo Integrativa da Literatura), conhecer as intervengóes de enfermagem na identificagáo, prevengáo e controlo do delirium no paciente adulto/idoso crítico.

## Materiais e Métodos

Quanto ao tipo de estudo, optou-se por uma RIL que permite obter várias perspetivas sobre um fenómeno, através de várias metodologias, sendo sintetizadas, com potencial para aplicagáo na prática baseada na evidencia, formando a base para a prática de enfermagem^8^^-^^9^.

Foi formulada a seguinte questáo de investigagáo: Quais as intervengóes de enfermagem na identificagáo, prevengáo e controlo do delirium no paciente adulto/idoso crítico?

Os critérios de selegáo foram definidos de acordo com a metodologia PI(C)CO8 (acrónimo para populagáo, intervengáo, comparagáo, contexto e resultados): Populagáo (adultos, com idade >18 anos), Intervengáo (intervengóes de enfermagem), Contexto (cuidados críticos), Comparagáo (náo se aplica), Outcome (identificar as intervengóes de enfermagem na prevengáo do delirium).

Estratégia de busca: para a realizagáo desta pesquisa foram usadas duas bases de dados electrónicas: a EBSCOhost (CINAHL Complete, MEDLINE Complete, Nursing & Allied Health Collection: Comprehensive, Cochrane Central Register of Controlled Trials, Cochrane Database of Systematic Reviews, Cochrane Methodology Register, Library, Information Science & Technology Abstracts, MedicLatina) e a B-on; durante o mes de Junho 2019.

Dois revisores independentes realizaram a avaliagáo crítica, extragáo e síntese dos dados. A leitura, bem como a avaliagáo da qualidade metodológica dos estudos, foi realizada para garantir a avaliagáo crítica durante o processo de selegáo dos artigos sendo que, perante algumas discordancias entre os pesquisadores foi pedida a avaliagáo de um terceiro avaliador. Todos os estudos apresentaram elevada qualidade pelo que náo foi excluído nenhum após esta avaliagáo.

A Qualidade Metodológica (QM) dos estudos foi efetuada através dos instrumentos do Joanna Briggs Institute - MAStARI- Checklist Test Accuracy Studies^9^.

Foi definido, previamente a realizagáo do estudo por todos os investigadores, que só se incluiriam os estudos com QM elevada, ou seja, que apresentassem um escore de 7, 8, 9 ou 10 no MAStARI^9^.

O acesso foi online, nas duas bases de dados. Os descritores usados e validados no MesH (Medical Subject Headings), foram os seguintes: Delirium, Nursing, Nursing Care, Critical Care, Patient, Child.

Foram Utilizadas para a selegáo de artigos, as seguintes combinagóes na língua inglesa, com os respetivos operadores booleanos (AND, NOT) e operador de truncamento (*): na primeira pesquisa foi utilizada a seguinte estratégia: Delirium (TI TITLE) AND “Critical Care” AND Patients AND Nursing NOT Child*. Na pesquisa seguinte: Delirium (TI TITLE) AND “Critical care” AND Nursing NOT Child*. Na terceira pesquisa:

Delirium (TI TITLE) AND “Critical care” AND Patients AND Nurs* NOT Child*. Na última pesquisa: Delirium (TI TITLE) AND “nursing care” AND “critical care” NOT child* (Tabela 1).

**Tabela 1:** Estratégia de Pesquisa

Pesquisa 1 (17/6)		Pesquisa 2 (17/6)		Pesquisa 3 (19/06)		Pesquisa 4 (19/6)	
Delirium (TI TITLE) AND “Critical care” AND Patients AND Nursing NOT Child*		Delirium (TI TITLE) AND “Critical care” AND Nursing NOT Child*		Delirium (TI TITLE) AND “Critical care” AND Patients AND Nurs* NOT Child*		Delirium (TI TITLE) AND “nursing care” AND “critical care” NOT child*	
EBSCOhost	B-ON	EBSCOhost	B-ON	EBSCOhost	B-ON	EBSCOhost	B-ON
Resultados: 436 Resultados (após limitadores: 86 (após remocáo de duplicados 59)	Resultados: 676 Resultados (após limitadores: 345 (após remocáo de duplicados 215)	Resultados: 543 Resultados (após limitadores:104 (após remocáo de duplicados 74)	Resultados: 948 Resultados (após limitadores: 447 (após remocáo de duplicados 303)	Resultados: 503 Resultados (após limitadores: 96 (após remocáo de duplicados 65)	Resultados: 801 Resultados (após limitadores:387 (após remocáo de duplicados 229)	Resultados: 31 Resultados (apólimitadores:12 (após remocáo de duplicados 12)	Resultados: 34 Resultados (após limitadores: 31 (após remocáo de duplicados 23)

Os critérios de inclusáo para a selegáo dos artigos foram os seguintes: acesso ao texto completo; artigos com data da publicado entre 2014 e 2019, artigos publicados no idioma portugués e inglés; estudos realizados em populagáo com idade > 18 anos e todos tipos de publicagóes. Numa fase inicial a selegáo comegou com a leitura dos títulos, seguida dos resumos, e por fim do texto integral, no sentido de responder a questáo de investigagáo. Os dados validados foram exportados e armazenados e com acesso público no Mendeley DataSet^10^.

## Resultados

**Figura 1:**
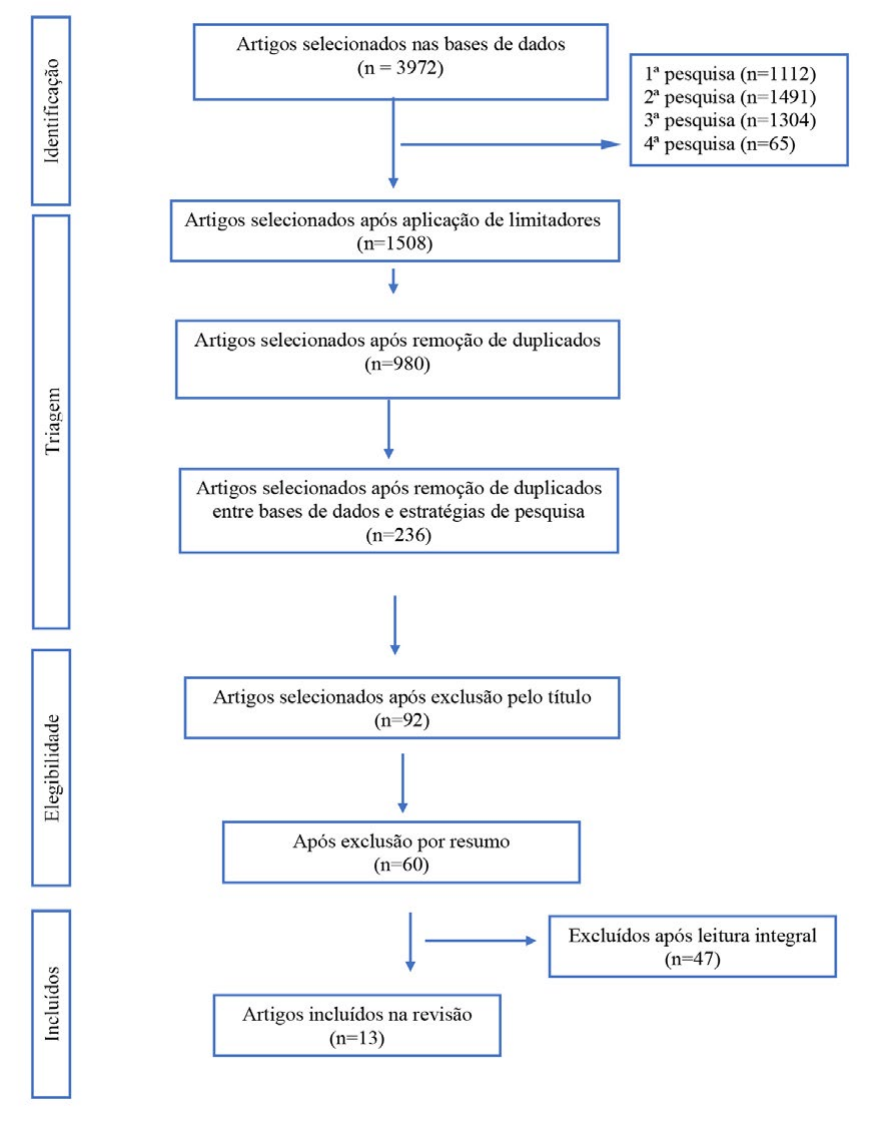
Fluxograma Prisma

Após a aplicagáo dos critérios de inclusáo e exclusáo foram selecionados 13 artigos que representam a amostra, sendo que correspondem a base de dados EBSCOhost. (Fig. 1 - Fluxograma Prisma).

Quanto ao ano de publicagáo dos 13 artigos, 1 foi publicado em 2013^4^, 3 em 2015^11^^-^^13^, 3 em 2016^14^^-^^16^ 3 em 2017^17^^-^^19^ e 3 em 2018^20^^-^^22^.

Relativamente ao país de origem dos estudos, 5 sáo dos EUA^11^^,^^14^^,^^18^^-^^20^, 1 da República da Coreia do Sul^12^, 1 do Chile^17^, 1 do Iráo^21^, 2 da Colombia^4^^,^^15^, 1 da República da Coreia^22^, 1 da Turquia^16^ e 1 do Brasil^13^.

Em relagáo desenho dos estudos, estes sáo: qualitativo observacional prospetivo^11^, retrospetivo^14^, randomizado controlado^12^^,^^18^^,^^20^^-^^21^, quase-experimental prospetivo^15^^-^^17^, coorte controlado^19^, revisáo sistemática e Meta-Análise^22^, revisáo da literatura^4^ e, revisáo integrativa da literatura^13^.

Procedeu-se a extragáo dos dados tendo por base as orientagóes do Joanna Briggs Institute de 2014^23^^)^ considerando: autor, ano e país; tipo de estudo, amostra e contexto; objetivos, conceito/ intervengóes; resultados e conclusáo. Os itens foram compilados numa tabela, com a finalidade de proceder ao resumo narrativo dos dados (Tabela 2).

**Tabela 2:** Análise dos artigos selecionados

Autor/Ano/País	Tipo Estudo/ Amostra/Contexto	Objetivo do Estudo	Nível Evidencia e Grau	Conceito/Intervencoes	Resultados/Conclusoes
Henao-Castaño ÁM, Amaya-Rey MCDP (2014^4^ Colombia	Revisáo da literatura. 47 artigos incluídos. Pacientes adultos internados em UTI na Colombia	Analisar a producáo científica acerca do delirium em pacientes em unidades de cuidados intensivos	4- C/QM=7	Análise de artigos selecionados que documentam a realizado do diagnóstico de delirium através de uma avaliacáo objetiva com instrumentos validados e estraté- gias para a prevencáo do mesmo.	Recomendam a interrupcáo diária da sedado. A demencia é o principal fa- tor de risco. O papel dos enfermeiros na prevendo passa pela avaliacáo do comportamento e estado mental dos pacientes e as intervendes de enfer- magem devem ser ajustadas a cada paciente, sendo importante detetar na anamnese fatores de risco.
Barros MAA de, Figueiredo DST de O, Fernandes M das GM, Neto JMR, Macedo-Costa KN de F (2015)^13^ Brasil	RIL: 16 artigos incluídos. Idosos em UTI.	Realizar um levantamento da literatura científica acerca do delirium em idosos em UTI	4- C/QM=9	Analise e síntese de estudos pu blicados com o intuito de com- preender que estudos tem sido realizados relativamente ao deli rium no idoso bem como estraté- gias de prevencáo em UTI	Verificou-se que a ocorrencia de de lirium em idosos em UTI está direta- mente ligada aos fatores de risco pre disponentes presentes nesta populado que náo sáo modificáveis, como carac terísticas pessoais e co-morbilidades. As intervendes náo farmacológicas e a utilizado de escalas de avaliacáo de delirium, demonstraram eficácia.
Rivosecchi RM, Kane-Gill SL, Svec S, Campbell S, Smithburger PL (2015^11^; EUA	Estudo qualitativo observacional prospetivo. 483 pacientes incluídos, na UTI do Centro Médico da Universidade de Pittsburgh- Hospital Presbyterian.	Determinar a eficácia da implementacáo de um protocolo náo farmacoló gico, na redujo do tempo de delirium na UTI.	2B- B/ QM=9	Avaliar a presenta ou predispo- sido a fatores de risco para o desenvolvimento de delirium, e avaliar a ICDSC. Protocolo de in- tervencoes náo farmacológicas - Protocolo “MORE”.	A implementado do protocolo de pre vendo de delirium náo farmacológi co, reduziu a o tempo de delirium dos pacientes em UTI (50,6%); reduziu a incidencia de delirium; reduziu o risco de desenvolvimento de delirium (57%).
Moon K-J, Lee S-M (2015)^12^; República Coreia do Sul	Estudo Randomizado Controlado. 123 pacientes incluídos com hospitalizado	Determinar os efeitos da aplicado de um protocolo preventivo de delirium, em pacientes de UTI, ana lisando seus efeitos sobre a incidencia de delirium, mortalidade intra-hospi- talar, readmissáo em UTI e tempo de permanencia na UTI.	1B- A/QM=10	Aplicado do protocolo de pre vendo de delirium com 4 com ponentes: 1 - Monitorizado e triagem do delirium: alteracoes cognitivas; alteracoes sensoriais e alteracoes físicas. 2- Avaliacáo cognitiva e orientado: CAM- ICU; fornecer informado acerca do ambiente e razáo da admissáo. 3- Intervencoes relacionadas com o ambiente. 4- Intervendo tera- peutica precoce.	A aplicado do protocolo reduziu a incidencia da mortalidade intra-hos- pitalar em 7 dias, mas náo teve efei- to sobre a incidencia de delirium. O papel do enfermeiro é importante na aplicado do protocolo, visto serem os profissionais de saúde que estáo 24h com do paciente, e assim prevenir o delirium, detetar precocemente e for- necer a intervendo precoce necessária. O programa de prevendo do delirium deve ser considerado uma atividade de enfermagem.
Bounds M, Harte S, Kram S, Daniel MG, Speroni KG, Brice K, et al (2016)^14^; EUA.	Estudo retrospetivo. 159 pacientes admitidos em 2 UTI Cirúrgicas e Médicas da Universidade de Maryland Shore Regional Health, em Dorchester e Easton.	Quantificar a prevalencia e durado do delirium em pacientes internados na UTI antes e após a implementado da Bundle ABCDE.	2B- B/QM=9	Implementar a Bundle ABCDE: A- Teste redudo de sedado diária, nos pacientes sob venti lado mecánica. B- Teste de respi rado espontánea, a cada 24 ho ras. C- Coordenado de testes de redudo de sedado e respirado espontánea; escolha de analgesia e sedado. D- Prevencáo e gestáo do delirium: uso da ICDSC; uso intervendes náo farmacológicas. E- Mobilizado precoce. F-Em- poderamento familiar.	A prevalencia de delirium diminuiu significativamente (de 38% para 23%) assim como o número médio de dias de delirium diminuiu de 3,8 para 1,72 dias, e o número de pacientes sem de lirium aumentou (62% para 77%) após a implementado da bundle. A bundle ABCDE pode ser eficaz na otimizacáo e melhoria na prestado de cuidados.
Tovar L, Suarez L, Muñoz F (2016)^15^; Colombia	Estudo qualitativo pré- experimental prospetivo. 49 pacientes incluídos, na UTI do Hospital Universitário de Neiva- Colombia	Determinar a eficácia dos cuidados prestados, de acordo o modelo de Betty Neuman), para controlar fatores precipitantes de delirium em UTI.	1B- A/QM=8	Aplicado do Guia de Enferma- gem em conjunto com as escalas RASS (Richmond Agitation Se dation Scale) e CAM-ICU. Con trolo dos fatores precipitantes ambientais para desenvolvimento do delirium.	Os cuidados de enfermagem de acordo com o Modelo de Betty Neuman, previ- nem o delirium em 94% dos pacientes, apesar da presenta de fatores de risco. enfermagem para a prevendo do deli rium em UTI.
Johnson K, Fleury J, McClain D (2018)^20^; EUA	Estudo randomizado controlado. 40 pacientes incluídos, na UTI Trauma e Unidade Trauma Ortopédico- Hospital em Phoenix- Arizona.	Avaliar a intervengo mu- siterapia para a prevengo do delirium nos pacientes internados na UTI de Trauma e UTI de Trauma Ortopédico; através da diminuido de variáveis fisiológicas: Pressao Arterial Sistólica (PAS), frequencia cardíaca (FC) e Frequencia respiratória (FR).	1B- A/QM=10	-Avaliacáo do CAM-ICU. O grupo de intervengo (recebeu phones e iPod numerados) foi su- jeito a musicoterapia durante 60 minutos, música pré-selecionada (estilo: Sintetizador; Harpa; Pia no; Orquestra; Jazz), duas vezes por dia, as 14h00 e as 20h00 tres dias após a admissáo, com tom baixo, ritmo lento e repetitivos para alterar respostas fisiológicas.	Verificou-se diminuido da FC, FR, PAS no grupo de intervendo. A músi ca aborda mecanismos fisiopatológicos que contribuem para o delirium, tais como: desequilíbrio de neurotransmis sores, inflamado e estressores fisioló gicos agudos. A música forneceu um suporte para prevenir o delirium de forma eficaz e seguro, em ambiente de terapia intensiva.
Hamzehpour H, Valiee S, Majedi M, Roshani D, Seidi J (2018)^((^^21^^))^; Iráo	Estudo Randomizado Controlado, triplo cego. 100 pacientes incluídos, em 2 UTI- Hospital Besat, Sanandaj, Irao.	Avaliar o efeito do plano de cuidados baseado no modelo de adaptado de Roy sobre a incidencia e gravidade do delirium em pacientes em UTI.	1B- A/QM=10	- Prestada formacáo de acordo com o Modelo de Roy aos enfer- meiros. Consiste em converter o comportamento desadaptativo (delirium) em comportamento adaptativo em sete dimensoes fi siológicas: equilíbrio hidroeletro- lítico, nutricáo, sono, atividade e mobilidade, excrecáo, condicoes de oxigénio e circulado e regu lado endócrina.O delirium foi medido pela NEECHAM Confu sion Scale.	- O plano de cuidados baseado no mo delo de adaptado de Roy reduziu a in cidencia e a gravidade do delirium. A organizado de diferentes intervencoes de enfermagem, em conjunto com in- tervencoes médicas e farmacéuticas conseguiram diminuir a incidencia e a gravidade do delirium na UTI.
Kang J, Lee M, Ko H, Kim S, Yun S, Jeong Y, et al (2018)^22^; República da Coreia	Revisao Sistemática e Meta-Análise 35 estudos: 15 metanálises e 20 estudos coorte.	Classificar as intervencoes nao farmacológicas utilizadas para prevenir o delirium na UTI e avaliar a sua eficácia.	1A- B/QM=10	As intervencoes náo farmacoló gicas utilizadas para a prevengo do delirium, foram classificadas em 9 categorias: multicompo- nentes; ambiente físico; interru- p$áo diária da sedado; exercício precoce; educado do paciente; sistema de alerta automático; melhoria da hemodinámica ce rebral; participado da familia e protocolo de redudo da sedado.	As Intervencoes náo farmacológicas foram eficazes na redudo da durado e ocorrencia de delirium. As inter- vencoes náo farmacológicas, mais uti lizadas nos estudos analisados, foram as intervendes multicomponentes (45,7%) e ambientais (25,7%).

A classificagáo de Oxford Centre for Evidence Medicine foi Utilizada para delinear os Níveis de Evidencia Científica e o Grau de Recomendado^24^. Os estudos incluidos tém elevado nivel de evidencia.

A incidencia do delirium no paciente crítico, foi determinada em todos os estudos incluídos.

Relativamente as intervengóes de enfermagem para a prevengáo do delirium, que os estudos fizeram referencia foram: relacionadas com o ambiente, a promogáo do sono, a intervengáo terapéutica precoce; a avaliagáo cognitiva e orientagáo, a utilizagáo de protocolos, a participagáo de familiares e a formagáo de enfermeiros e ensino dos pacientes. As que estáo relacionadas com o ambiente surgiram em dez estudos - 76,9%^4^^,^^11^^-^^12^^,^^14^^-^^17^^,^^20^^-^^22^; a promogáo do sono em dez estudos - 76,9% ^4^^,^^11^^-^^12^^,^^14^^) (^^17^^,^^20^^-^^22^; a intervengáo terapeutica precoce em oito estudos - 61,5%^4^^,^^11^^-^^12^^,^^14^^-^^17^^,^^20^^-^^22^ e as relacionadas com a avaliagáo cognitiva e orientagáo dos pacientes em dez estudos- 76,9%^4,(^^11^^-^^18^^,^^22^. As intervengóes sistematizadas em protocolos surgiram em cinco estudos- 38,4%^11^^-^^12^^,^^14^^,^^19^^,^^22^.

A implementagáo da bundle ABDCE (Awakening and Breathing Coordination, Delirium Monitoring and Management e Early Mobility) diminuiu a prevalencia do delirium (de 38%-23%), o número de dias sem delirium (3,8- 1,72 dias) e o número de pacientes sem delirium (62%-77%). A bundle DPB (Delirium Prevention Bundle), foi eficaz na redugáo do delirium em 78%. A implementagáo da bundle “MORE” (Music, Opening of blinds, Reorientation e Cognitive stimulation, Eye and Ear protocol), evidenciou uma redugáo do delirium em 57%, redugáo tempo internamento em UTI (50,6%).

Numa revisao sistemática - Meta-análise^22^, 16 estudos fizeram referencia as intervengóes através de protocolos (45,7%), evidenciando a redugao e ocorrencia do delirium. O protocolo de prevengao^12^, demonstrou reduzir a mortalidade em sete dias, mas sem diminuigao significativa da incidencia do delirium.

As intervengóes dirigidas a participagao dos familiares^4^^,^^13^^-^^14^^,^^17^^,^^22^ surgiu incluida em cinco estudos - 38,4%, enquanto a formagao dos enfermeiros e ensino dos pacientes em tres estudos - 23%^11^^,^^16^^,^^22^.

Deste modo, constatou-se que as intervengóes que surgiram com mais frequencia nos estudos selecionados foram as relacionadas com o ambiente, promotoras do sono e avaliagao cognitiva e orientagao dos pacientes.

**Tabela 3:** Intervengóes de Enfermagem na Prevencáo do Delirium

Componente	Intervengóes de Enfermagem
Ambiente	Providenciar abertura/ encerramento das persianas.
	Facilitar musicoterapia, em tom baixo, ritmo lento e repetitivos.
	Providenciar óculos e aparelhos auditivos, caso alterado da acuidade visual e auditiva.
	Providenciar ambiente harmonioso: proximidade com os enfermeiros, objetos familiares, minimizar movimentacóes do paciente, reduzir volume dos alarmes do monitor e telefo nes, aromaterapia.
Promodo sono	Proporcionar massagens relaxantes e posicionamento confortável.
	Facilitar luz adequada ao ciclo circadiano e redudo do ruido.
	Providenciar, se necessário, indutores do sono.
	Evitar que a pessoa durma durante o dia e evitar procedimentos durante a noite.
	Providenciar locais de menor ruído para doentes mais fragilizados. Proporcionar tampóes para os ouvidos (se necessário).
Intervengo terapéutica precoce	Providenciar nutrido, hidratado e oxigenado adequadas.
	Prevenir situacóes de hipo/hipertermia e hipo/hiperglicémia.
	Proporcionar mobilizacao precoce e exercício.
	Proporcionar fármacos indutores do sono/sedativos adequados a prevendo do delirium precocemente.
	Remover cateteres desnecessários e despistar sinais precoces de infecao.
	Proporcionar um controlo adequado da dor.
	Monitorizar eliminado vesical e prevenir obstipacao precocemente.

As intervengóes identificadas podem ser organizadas em tabela da seguinte forma:

No que se refere aos fatores de risco potenciadores do delirium, foram incluídas as alteragóes cognitivas, alteragóes sensoriais e alteragóes físicas em cinco estudos- 38,4%^4,(^^12^^,^^13^^,^^16^^,^^21^.

Para a avaliagao do delirium dos pacientes em UTI, os estudos selecionados abordaram os seguintes instrumentos: ICDSC (Intensive Care Delirium Screening Scale Checklist)^11^^,^^14^^,^^22^, CAM-ICU^4^^,^^12^^-^^13^^,^^15^^-^^20^, NEECHAM Confusion Scale^21^^-^^22^, DDS (Delirium Detection Score)^22^, DOS (Delirium Observation Screening Scale)^22^, DSM IV (Diagnostic e Statistical Manual of Mental Disorders IV)^22^. Verificou-se que na maioria dos estudos, ou seja em 69,2%, é usada a CAM- ICU.

## Discussao

Nos pacientes críticos, o desenvolvimento do delirium está associado a várias consequéncias negativas, como o aumento do tempo de internamento, da mortalidade e morbilidade, assim como ao elevado custo hospitalar. Torna-se assim importante e imperativo a prevengao da ocorréncia do delirium^25^^-^^26^.

O conhecimento e a identificagao precoce dos fatores de risco na admissao do paciente e nas primeiras 24 horas por parte dos enfermeiros para o desenvolvimento do delirium e seu diagnóstico, sao importantes para a aplicabilidade de medidas profiláticas e de tratamento, uma vez que conseguem predizer a ocorréncia do delirium^7^. Assim, devem estar incluídos no algoritmo das intervengóes de enfermagem na prevengao do delirium o reconhecimento dos seguintes fatores de risco: alteragóes cognitivas (dificuldade na concentragao, confusao, irritabilidade), alteragóes sensoriais (alteragóes da acuidade visual e auditivas) alteragóes físicas (mobilidade reduzida, agitagao, distúrbios do sono, alcoolismo, tabagismo, distúrbios hidroeletrolíticos, hipo/hipertermia, anemia, alteragóes fungao renal, anemia) e alteragóes sociais (dificuldade na comunicagao, alteragóes do humor, incapacidade de colaboragao)^4^^,^^12^^,^^13^^,^^16^^,^^21^.

Além do reconhecimento dos fatores de risco acima descritos, os enfermeiros tornam-se responsáveis no controlo de fatores de risco potencialmente modificáveis, tais como: promogao da nutrigao e hidratagao, gestao de dispositivos clínicos, promogao de visitas de familiares, promogao da Utilizagao de próteses auditivas e dentárias, gestao adequada da medicagao prescrita, posicionamentos e oxigenoterapia adequados^27^.

Após análise do conteúdo dos estudos, constatou-se que existem várias intervengóes de enfermagem, que podem ser agrupadas em sete categorias: o ambiente; promogao do sono; intervengao terapéutica precoce; avaliagáo cognitiva e orientado dos pacientes; protocolos; participado dos familiares; formado dos enfermeiros e ensino do paciente (Tabela 3).

As ades de enfermagem relacionadas com o ambiente, sáo aquelas que promovem um ambiente acolhedor e confortador, com o enfermeiro de referencia sempre próximo, possibilitam o uso de objetos pessoais, a presenta de familiares, procedem a deslocado do paciente apenas quando necessário, reduzem o volume dos alarmes dos dispositivos e telefones (para diminuir a ansiedade) e providenciam a abertura/encerramento das persianas para ambiente confortador^4^^,^^11^^-^^12^^,^^14^^-^^17^^,^^20^^-^^11^.

A musicoterapia, demonstra ser eficaz na prevendo porque aborda mecanismos que contribuem para o delirium, como o desequilibrio de neurotransmissores, inflamado e estressores fisiológicos agudos. Verificou-se a diminuido de variáveis fisiológicas (frequencia cardíaca, frequencia respiratória e pressáo arterial); regulado do stress e emogóes, com composigóes musicais suaves (ritmo lento, tom baixo, ritmos repetitivos) e com isso diminuigáo do delirium^20^.

Dada a elevada incidencia do delirium nas UTI's, a prevengáo tem um papel preponderante e está associada a uma elevada eficácia, baixo risco e baixo custo^25^. Um conjunto de intervengóes, ou seja, protocolos, sáo mais eficazes que intervengóes isoladas^7^, nas quais a participagáo do enfermeiro é de elevada importancia.

O protocolo MORE^11^: música; abertura/encerramento de persianas, reorientagáo/ estimulagáo cognitiva (uso de calendário, entretenimento visual), demonstrou uma redugáo do tempo de internamento e do desenvolvimento de delirium.

Além deste protocolo, também houve referencia a bundle ABCDE^14^, que consiste no teste de redugáo de sedagáo; teste de respiragáo espontanea; coordenagáo com outros membros da equipa multidisciplinar; escolha da sedagáo e analgésico; prevengáo e avaliagáo do delirium; mobilizagáo precoce.

Este estudo sugeriu a inclusáo do empowerment familiar, como estratégia de prevengáo do delirium, dando origem ao ABCDEF. Com estas medidas implementadas demonstrou-se uma diminuigáo da prevalencia e duragáo do delirium. Tal, foi comprovado num outro estudo, realizado em sete hospitais da Califórnia, com uma amostra de 6024 pacientes, internados em UTI^28^.

Neste contexto foi implementada a PDA Guidelines (Clinical Practice Guidelines for the Management of Pain, Agitation, and Delirium in Adult Patients in the Intensive Care Unit), através da bundle ABCDEF, e foi possível demonstrar redugáo significativa da mortalidade, do delirium e coma. Esta bundle foi reformulada, acrescentando dois itens: avaliagáo/tratamento da dor e empowerment familiar.

Através da suspensáo da sedagáo, controlo da dor, estimulagáo sensorial, mobilizagáo precoce e promogáo do sono, que correspondente a bundle DPB^19^, foi possível demonstrar uma redugáo efetiva do delirium. Para além da diminuigáo do delirium, verificou-se a diminuigáo da incidencia da mortalidade em sete dias, quando implementado um protocolo que consiste na verificagáo dos fatores de risco, avaliagáo do delirium e intervengóes relacionadas com o ambiente e intervengáo terapeutica.

Existem outras investigagóes em curso, com um delineamento de um protocolo de intervengóes de enfermagem para a prevengáo do delirium, o UNDERPIN-ICU (Delirium Interventions in the Intensive Care Unit)^29^, que consiste na promogáo da adequada fungáo auditiva e visual, que quando comprometidas os cuidados devem ser adaptados, promogáo do sono adequado e otimizagáo ciclo circadiano, estimulado cognitiva, estimulado da mobilizagáo e redudo da sedado.

Os pacientes internados em UTI, sofrem alterado no seu padráo de sono, em consequéncia do ruido, da luz e de todas as várias intervendes que sáo sujeitos^30^. Os enfermeiros devem minimizar esses fatores, devendo desenvolver agóes promotoras do sono. Estáo incluidas a aromaterapia; massagens relaxantes (pés e costas); luz adequada ao ciclo circadiano; náo interromper o sono; redugáo ruido; comprimidos indutores do sono. Sempre que possivel realizar procedimentos durante o dia, providenciar o posicionamento confortável e preferido para o paciente; pacientes mais fragilizados, colocar em ambiente com menos ruido^4^^,^^12^^,^^14^^-^^17^^,^^21^^,^^22^.

Neste contexto, um protocolo noturno de redugáo de som (uso de tampóes durante a noite; redugáo volume alarmes; limitar o número de interrupgóes do sono e quando sujeitos a técnica substituigáo renal continua retirar os sacos das solugóes, afastados do paciente) permitiu reduzir a incidéncia de delirium em pacientes internados na UTI^31^^-^^32^.

Em relado a intervendo terapéutica, verificou-se que a nutrido e hidratado adequadas, prevengáo de hipo/hipertermia e hipo/hiperglicémia, oxigenagáo adequada, mobilizagáo precoce e exercicio, revisáo permanente dos fármacos indutores do sono/sedativos, despistar sinais precoces de infegáo, remogáo de cateteres desnecessários, controlo da dor, evitar hipoxia, monitorizagáo da eliminagáo vesical e prevenir obstipagáo, foram eficazes na prevengáo do delirium^4,(^^11^^-^^12^^,^^14^^,^^16^^,^^17^^,^^21^^-^^22^^).^

A avaliagáo cognitiva e orientado dos pacientes, sáo de extrema importancia, uma vez que as UTI's sáo locais propensos a momentos de stress, pela gravidade dos pacientes e náo deve ser descurada a orientagáo quanto ao local e suas caracteristicas, identificagáo do profissional, data, hora e razáo pela qual está na UTI, permissáo de visitas de familiares e amigos, providenciar leitura, quando aplicável, fornecimento de calendários aos pacientes e relógios com monitor de elevadas dimensóes.

Uso de comunicagáo verbal e náo verbal adequados. Minimizar as restrigóes fisicas, usar apenas temporariamente perante quadros de agitagáo severa, para protegáo do paciente^4^^,^^12^^-^^13^^,^^15^^-^^18^^,^^22^. A incidéncia do delirium nos pacientes em UTI é elevada com o uso de restrigóes fisicas e o uso destas medidas deve ser minimizado^33^.

Um outro aspeto referenciado é o fornecimento de orientagáo na UTI através de mensagens áudio com voz familiar, verificou a sua eficácia na redugáo do delirium. Estas devem conter informagóes de orientagáo espago/temporal, frases confortadoras e que tranquilizem o paciente. A facilidade de implementagáo associada a baixo custo, torna esta estratégia eficaz na prevengáo do delirium^18^.

A participado dos familiares refere-se a inclusáo de membros da familia na prestado de cuidados ao paciente na UTI, melhorando a comunicagáo entre pacientes, familiares e equipa multidisciplinar, aumentado a confianga do paciente^34^. Incluir os membros da familia a fazer parte da estratégia de estimulagáo do paciente e permitir o horário alargado de visitas, tém evidéncia na prevengáo do delirium e promove o empowerment familiar^13^^,^^14^^,^^17^^,^^22^.

A prevengáo do delirium passa pelas intervengóes náo farmacológicas, tais como a reorientagáo, estimulagáo cognitiva, promogáo de sono de qualidade, redugáo da imobilidade, providenciar dispositivos no caso de incapacidade auditiva e visual^7^.

O uso instrumentos de avaliagáo de delirium, facilitam o seu reconhecimento precoce, permitindo adotar medidas no sentido da sua prevengáo e diminuido da gravidade. As escalas abordadas nos estudos, foram as seguintes: ICDSC (Intensive Care Delirium Screening Scale Checklist)^11^^,^^14^^,^^22^, CAM- ICU^4^^,^^12^^,^^13^^,^^15^^-^^20^^,^^22^, NEECHAM (Confusion Scale)21,22, DDS (Delirium Detection Score)^22^, DOS (Delirium Observation Screening Scale)^22^, DSM IV (Diagnostic e Statistical Manual of Mental Disorders IV)^22^.

A PADIS Guidelines de 2018^7^, recomenda o uso da CAM-ICU e ICDSC, sendo as que apresentam maior sensibilidade. Para a populagáo portuguesa é validada e usada a CAM-ICU^6^, sendo um instrumento com elevada sensibilidade, especificidade e simples de utilizar.

O desconhecimento acerca dos tipos de delirium, das manifestares e sua prevengáo, levam a maior probabilidade de ocorréncia do delirium. Assim torna-se importante a formagáo dos enfermeiros acerca da fisiopatologia, monitorizagáo, fatores de risco, escalas de avaliagáo e intervengóes náo farmacológicas do delirium; ensinos ao paciente acerca das intervengóes que váo ser efetuadas, e o que é expectável acontecer, tranquilizando o paciente^11^^,^^16^^,^^22^.

Apresenta-se como uma limitagáo deste estudo a possibilidade de náo ter esgotado todos os artigos publicados sobre este assunto. Além disso, a literatura sobre o tema ainda está com baixo grau de evidéncias, o que mostra a necessidade de estudos mais aprofundados.

## Conclusao

Esta RIL possibilitou conhecer as intervengóes de enfermagem na identificagáo, prevengáo e controlo da ocorréncia do delirium, no paciente crítico.

O enfermeiro deve ter conhecimento dos instrumentos de avaliagáo do delirium, assim como as suas manifestagóes e fatores de risco relacionados, podendo assim atuar no sentido de prevenir e diminuir a sua ocorréncia e gravidade. Sáo intervengóes com facilidade de implementagáo, baixo custo e comprovada elevada eficácia.

Com este estudo concluiu-se que o enfermeiro tem um papel muito importante na prevengáo do delirium do paciente crítico, com a adogáo de medidas relacionadas com o ambiente, promogáo do sono, intervengáo terapéutica, avaliagáo cognitiva e orientagáo dos pacientes, protocolos, participagáo dos familiares, formagáo dos enfermeiros e ensino do paciente.

A implementagáo deste conjunto de medidas, é mais eficaz na diminuigáo da ocorréncia do delirium, comparadas com o seu uso de forma isolada.

Sugere-se a realizagáo de mais estudos, para a comprovagáo da eficácia destas medidas, em particular na populagáo portuguesa em UTI.

Os resultados deste estudo contribuem para a melhoria do cuidado em saúde, concretamente na prática de enfermagem no que respeita a prevengáo e controle do delirium, contribuindo para a diminuigáo da mortalidade e morbilidade do paciente adulto/idoso crítico. Em prol de uma enfermagem avangada, reconhece-se a importancia da sensibilizagáo dos enfermeiros e restante equipa para o avango do conhecimento nesta matéria.
